# Physico-Chemical Surface Modifications of Polyetheretherketone (PEEK) Using Extreme Ultraviolet (EUV) Radiation and EUV-Induced Nitrogen Plasma

**DOI:** 10.3390/ma13194466

**Published:** 2020-10-08

**Authors:** Joanna Czwartos, Bogusław Budner, Andrzej Bartnik, Przemysław Wachulak, Henryk Fiedorowicz, Zygmunt Mierczyk

**Affiliations:** Institute of Optoelectronics, Military University of Technology, 2 Kaliskiego St., 00-908 Warsaw, Poland; boguslaw.budner@wat.edu.pl (B.B.); andrzej.bartnik@wat.edu.pl (A.B.); przemyslaw.wachulak@wat.edu.pl (P.W.); henryk.fiedorowicz@wat.edu.pl (H.F.); zygmunt.mierczyk@wat.edu.pl (Z.M.)

**Keywords:** PEEK, surface modifications, surface chemistry, XPS analysis, AFM, extreme ultraviolet (EUV), low-temperature plasma

## Abstract

In this work, the effect of extreme ultraviolet (EUV) radiation and the combination of EUV radiation and low-temperature nitrogen plasma on the physico-chemical properties of polyetheretherketone (PEEK) surfaces were presented. The laser-plasma EUV source based on a double gas puff target was used in this experiment to irradiate PEEK surfaces with nanosecond pulses of EUV radiation and to produce low-temperature plasma through the photoionization of nitrogen with EUV photons. The changes in surface morphology on irradiated polymer samples were examined using atomic force microscopy (AFM) and scanning electron microscopy (SEM). Chemical changes of the PEEK surfaces were analysed using X-ray photoelectron spectroscopy (XPS). EUV radiation and nitrogen plasma treatment caused significant changes in the topography of modified PEEK’s surfaces and an increase in their average roughness. Strong chemical decomposition, appearance of new functional groups as well as incorporation of nitrogen atoms up to ~17 at.% on the PEEK’s surface were observed.

## 1. Introduction

Polyetheretherketone (PEEK) is a high-temperature, thermoplastic synthetic polymer that belongs to the polyaryletherketones (PAEK) family. The polymer is a very interesting material due to its excellent physico-chemical properties. PEEK has good thermal stability, good chemical resistance [1], and is non-toxic as well as biocompatible; it has also excellent mechanical properties, including strength, stiffness, toughness [2] and is transparent to X-rays. Due to these properties, there are multiple industries in which PEEK can be used, including automotive, aerospace, microelectronics, packaging, and, primarily, biomedicine, in which it is considered as a perfect substitute for metallic implant materials (bone surgery, jaw surgery, orthopedics) [3,4]. Despite the advantages mentioned, PEEK—similarly to many other organic polymers [5,6,7,8,9,10]—shows low bioactivity, is hydrophobic, and lacks surface-active functional groups that promote protein adsorption, cell adhesion, and proliferation. This is why PEEK, when implanted, shows limited integration with osseous tissues. It is the factor which strongly limits its clinical application. Thus, appropriate physico-chemical modification of the PEEK surface, and its micro- and nanostructuring, can improve its bioactivity. Many techniques were used for PEEK modification, including chemical surface treatment [11,12], laser processing [13,14], and the most popular, which seems also to be the most universal—surface plasma treatment [15,16]. The effect of the exposure to plasma induces a variety of changes on polymer surfaces. These include surface ablation or etching, crosslinking or chemical changes such as the incorporation of atoms or various polar functional groups. One of the major results of plasma polymer treatment is the changes in surface topography that affect roughness, wettability, and, consequently, adhesion and bioactivity. Examination of the influence of plasma treatment using different plasmas on PEEK physico-chemical parameters and its biocompatibility has been the subject of numerous studies. For example, Waser-Althaus et al. [17] observed that oxygen and ammonia plasma treatment of PEEK induced strong chemical changes (i.e., oxidation or implantation of surface-amino groups) and topography changes (i.e., nanostructuring). What is also important is that it supported the osteogenic differentiation of human mesenchymal stem cells (adMSC). On the other hand, Novotna et al. [18] modified the surface of PEEK using argon plasma and observed that exposure to plasma resulted in the improvement of PEEK’s wettability and caused changes in its surface morphology and chemistry. The changes in PEEK’s surface, induced this way, significantly enhanced mouse fibroblasts and human osteoblasts adhesion, proliferation, and also metabolic activity. Other studies exhibit that PEEK plasma treatment can considerably improve its adhesive features, which is very important when applying various organic or non-organic coatings [19,20,21,22].

In this study, an alternative technique of surface modification, using intense nanosecond pulses of extreme ultraviolet (EUV) for PEEK foils irradiation and the EUV irradiation combined with a low-temperature nitrogen plasma treatment, was presented. Polymer surfaces exposure to EUV radiation results in surface ablation, surface geometry changes, and modification of their chemical structure. Moreover, additional usage of the low-temperature plasma induced by the EUV beam in a gas injected into the area surrounding the irradiated polymer surface causes the incorporation of new functional groups into the polymer surface. In this paper, the detailed chemical analysis of modified PEEK foils carried out based on high-resolution XPS spectra analysis is presented. Changes in surface morphology were examined using atomic force microscopy (AFM) and scanning electron microscopy (SEM).

## 2. Materials and Methods 

### 2.1. Modifications of PEEK Films

**Figure 1:**
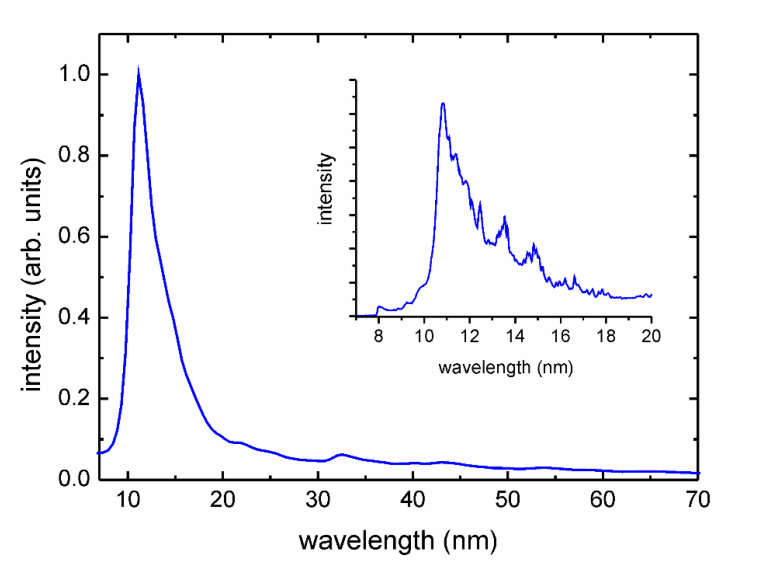
The spectrum of the extreme ultraviolet (EUV) radiation emitted from laser-produced plasma (LPP) Xe plasmas and focused by the ellipsoidal collector. In the inset, the most intense part of the spectrum, measured with higher resolution, is presented.

In the experiment, the amorphous PEEK films with a thickness of 0.075 mm (Goodfellow Cambridge Ltd., Huntingdon, UK) were modified. The modifications were performed using a laser-produced plasma (LPP) EUV source dedicated to the surface processing of polymers [23]. The source was developed in our laboratory. It is based on a gas-puff target system and a compact Nd:YAG laser producing 4 ns laser pulses with maximum energy up to 0.8 J (NL 303 HT, EXPLA, Vilnius, Lithuania) and 10 Hz repetition rate. In the experiment, the target was formed as a result of a pulsed injection of a xenon jet into a hollow stream of helium (Xe/He target) with the use of an electromagnetic valve system equipped with a double nozzle set-up. The laser beam focused on the Xe stream created the high-temperature plasma. In the experiment, focusing conditions and plasma parameters were adjusted in order to obtain a maximum intensity in extreme ultraviolet. The EUV radiation was focused using a gold plated grazing incidence ellipsoidal collector (RITE s.r.o., Dolni Břežany, Czech Republic). The collector allowed for effective focusing of Xe plasma radiation within the wavelength range of λ = 9–70 nm—Figure 1.

**Figure 2:**
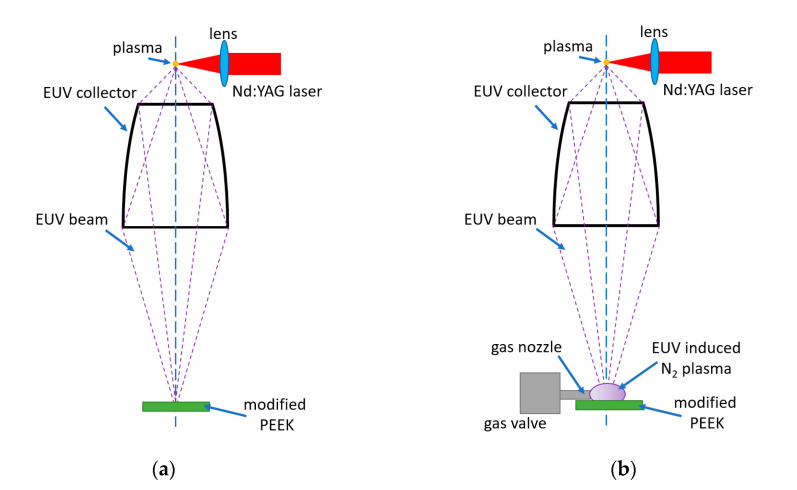
The scheme of experimental arrangement: (**a**) polyetheretherketone (PEEK) sample modified with EUV radiation, (**b**) PEEK sample modified with EUV radiation and nitrogen plasma.

The maximum intensity was in a relatively narrow spectral region centered at λ ≈ 11 nm. The EUV fluence in the focal plane of the collector was about 60 mJ/cm^2^ at the centre of the focal spot. A part of the EUV beam could be used for photoionization of the nitrogen gas injected through the auxiliary gas-puff valve into the region of the focal spot, perpendicularly to an optical axis of the EUV collector. Photoionization of the gas by the intense EUV radiation pulse resulted in a low-temperature plasma formation. Spectral measurements of the nitrogen plasmas, performed in the wide wavelength range (EUV-UV-VIS), revealed the presence of single charged atomic ions (N^+^), molecular ions (N_2_^+^), and also neutral atoms (N) and molecules (N_2_). A detailed description of the EUV-induced plasmas can be found elsewhere [24]. Nitrogen gas density in the interaction region was controlled by the adjustment of the gas-puff valve opening time [25]. The PEEK samples were mounted on the XYZ movable stage, located 3 mm from the focal spot and irradiated with 40 and 150 pulses at a 10 Hz repetition rate. The EUV irradiation of the polymer films was carried out without (a) and with (b) nitrogen presence—Figure 2. In the case of using the nitrogen, a part of the radiation was absorbed, ionizing the gas. The second part reached the polymer surface. This way, the dual action of plasma and the EUV radiation on the material surface was possible. 

The modified area on the polymer’s surface was circle-like shaped with a diameter of ~1.9 mm. Such a size of the modified surface was large enough for morphological examinations of the PEEK samples, performed using an atomic force microscope (AFM). For XPS studies to be performed, the area of ~0.5 × 0.7 cm^2^ (7 spots in total) was modified.

### 2.2. Surface Analysis of Modified PEEK Surfaces

#### 2.2.1. Atomic Force Microscopy (AFM) and Scanning Electron Microscopy (SEM)

Surface morphology, profile, and roughness of pristine and modified PEEK samples were examined using an AFM (NT-MDT Spectrum Instruments, Moscow, Russia). The measurements were carried out at ambient conditions in a semi-contact mode using a golden silicon AFM probe (NSG 10, NT-MDT Spectrum Instruments, Moscow, Russia) featuring a pyramidal tip with a curvature radius of ~10 nm. The cantilever of the probe was characterized by a force constant range from 3.1 to 37.6 N/m and a resonant frequency range of 140–390 kHz. The scan size of the topographies collected was 50 µm × 50 µm with a resolution of 256 points per line. Three different regions were scanned on each sample and the average surface roughness was calculated using Image Analysis software (version-3.5 provided by NT-MDT Spectrum Instruments.

SEM scans were captured with a scanning electron microscope (Quanta FEG250, FEI, Hillsboro, OR, USA). The acceleration voltage was 20 kV. All samples studied were deposited with a platinum layer of 8 nm thickness. The platinum layer was deposited from a platinum target (99.999%) by means of a sputter coater (Leica EM ACE200, Leica Microsystems, Wetzlar, Germany).

#### 2.2.2. X-ray Photoelectron Spectroscopy (XPS)

Chemical analysis of the PEEK surfaces modified with 40 and 150 pulses of EUV radiation and a combination of EUV radiation and low-temperature nitrogen plasma was performed using X-ray photoelectron spectroscopy (XPS). The spectrometer XPS (Prevac, Rogów, Poland) used was equipped with the SCIENTA R3000 analyser (VG Scienta, Uppsala, Sweden) and an X-ray lamp with the Al K_α_ anode (Prevac, Rogów, Poland). During the measurements, the pressure in the ultra-high vacuum chamber of the XPS system was approximately 3 × 10^−9^ mbar. Before the XPS examination, the non-modified PEEK foil was cleaned using ethyl alcohol, while the modified PEEK samples were examined without any cleaning. A thorough chemical analysis, i.e., high-resolution spectra in the narrow ranges of binding energy with a 40 meV step and pass energy of 100 meV for each band: C1s (294–282 eV), N1s (404.5–395.5 eV), and O1s (537–528 eV), were recorded. The peaks associated with the C1s, N1s, and O1s bands were fitted using CasaXPS software (version 2.3.23, www.casaxps.com). The background of linear type and Gaussian–Lorentzian (GL) line shape (GL 50 for C1s, GL 60 for N1s, and GL 55 for O1s) were fitted for all these bands. All XPS spectra measured were shifted in such a way that the maximum of the C–C=C peak (refers to carbon ring in PEEK’s mer unit) was at 284.8 eV.

## 3. Results and Discussion

### 3.1. Morphological Changes on PEEK Surfaces

**Figure 3:**
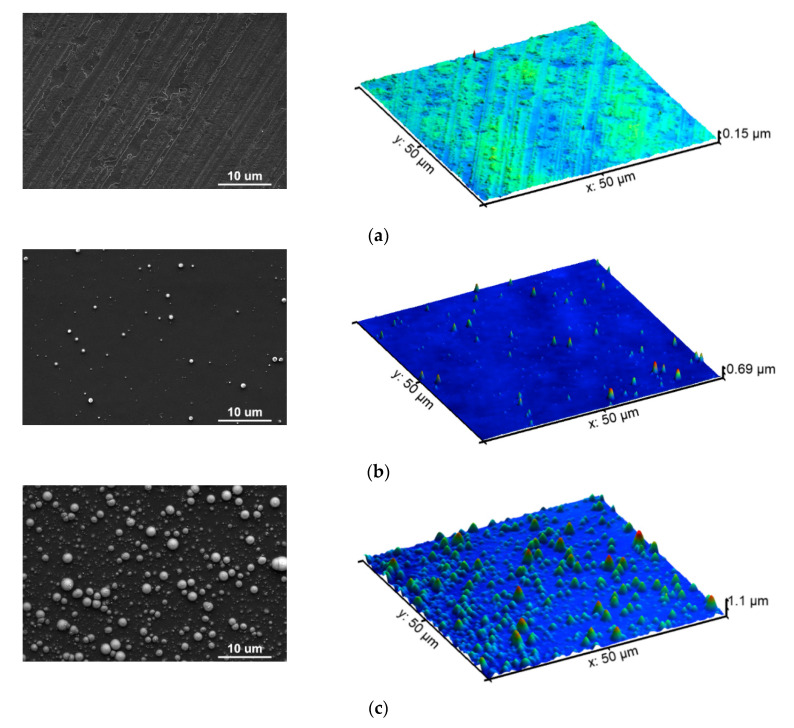
SEM images compared with the AFM topographies of (**a**) untreated PEEK, (**b**) PEEK treated with 40 EUV pulses, (**c**) PEEK treated with 150 EUV pulses.

EUV irradiation of untreated polymer foils results in the formation of different structures on their surfaces. The character of the topography obtained, i.e., the shape of the micro- and/or nanopatterns formed, their size, height, or directionality, strongly depends on the number of pulses used, fluence, and the physico-chemical properties of the pristine polymer, i.e., the material sensitivity, ablation threshold value, thermal conductivity, glass transition temperature, etc. In this experiment, the AFM was employed to examine the morphology of the PEEK surface before and after EUV irradiation. As simultaneous treatment of PEEK surfaces by EUV radiation and nitrogen plasma resulted in the formation of the same pattern as in the case of only PEEK-treated EUV radiation, the presentation of the results for that case was omitted. Figure 3 shows the AFM topographies of the non-modified PEEK surface and the PEEK surface modified with 40 and 150 EUV pulses with a scan size of 50 µm × 50 µm and with their corresponding SEM images in a similar scale. 

**Figure 4:**
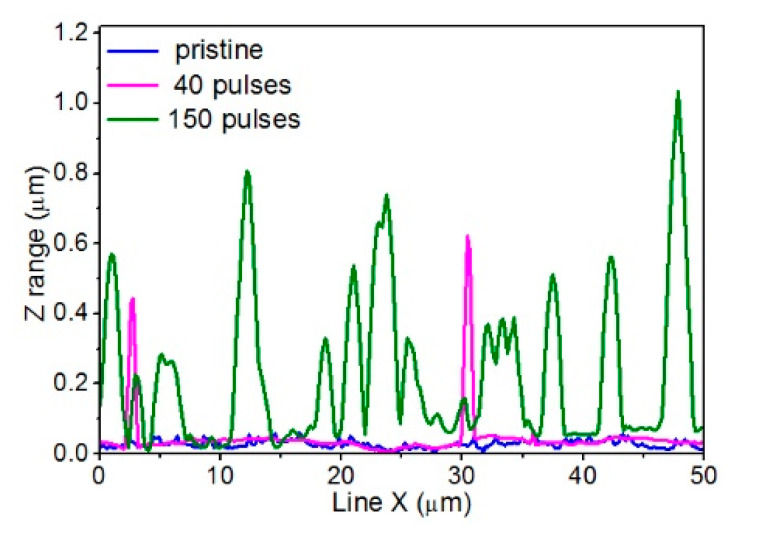
Cross-section profiles obtained from the AFM topographies along the selected line for untreated PEEK and PEEK treated with 40 and 150 EUV pulses, respectively.

As shown in the Figure 3, the unmodified PEEK surface originally had specific subtle trails in the form of grooves and irregularities running parallel to the foil’s edge in one direction. These trails most likely reflected irregularities on the surface of the mold or roller with which the PEEK foil got in contact during the production process. The average roughness calculated for this sample was 16.2 ± 2.0 nm. The height of the sample’s original structures measured using cross-section analysis of the AFM topographies (where the profile of the PEEK structure was obtained along with a single, selected line), did not exceed 60 nm—Figure 4 (blue line). 

The topography of PEEK treated with 40 EUV pulses changed significantly compared to the pristine PEEK. The specific trails running in one direction were not there anymore, and the surface was clearly ablated. Moreover, randomly located micro- and nanometer-scale features appeared on the surface. The larger features were conical in shape as evidenced by not only AFM scans but also the cross-section profiles obtained from the AFM topographies (see Figure 4—pink purple line). Their maximum diameter at the base was ~1 µm and the heights did not exceed 700 nm. Diameters of the smallest structures on the surface were approximately 100 nm. The average roughness measured for this sample increased, compared to the non-modified one, and was 45 ± 5 nm. 

The number and size of these features on the PEEK surface treated with 150 EUV pulses significantly increased. The maximum diameter of the conical structures at the base even exceeded 3 µm, while the diameters of the smallest structures were still ~100 nm. The heights of the cones on the surface exceeded 1 µm in some cases. The average roughness for this sample almost doubled compared to PEEK modified with 40 EUV pulses and reached 115 ± 11 nm. 

Therefore, the surface density of the features as well as the average roughness of the foil increased with the number of EUV pulses. 

The formation of conical structures on the PEEK surface is probably connected with the shielding effects related to local enrichments of photofragments or material impurities having a higher ablation threshold than the polymer [26] Smaller features with diameter about 100–200 nm, which are visible both on the surface of PEEK and at the top of the cones, may arise as a result of hydrodynamic instabilities, as suggested by Bauerle [26], and also be responsible for the formation of the conical structures [26,27,28]. Similar conical structures on the surfaces of various polymers (PET, PMMA, PTFE, FEP) irradiated with EUV pulses have been already observed by our group in previous experiments [29,30,31,32]. The same structures were also observed by other authors who irradiated surfaces of various organic polymers, including PC, PI, PET, nylon, etc., with UV light from excimer lasers [27,33].

### 3.2. Chemical Analysis of Pristine and Modified PEEK Surfaces

**Figure 5:**
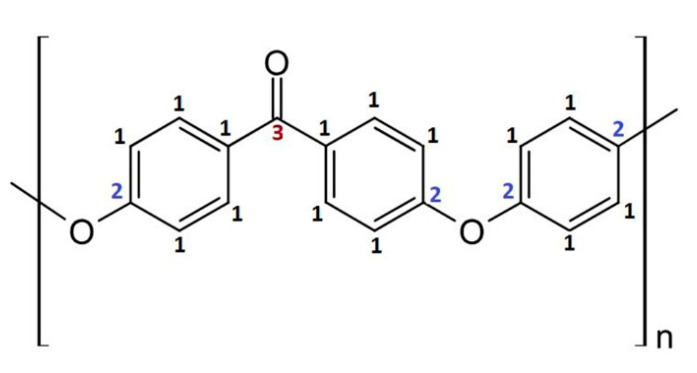
Chemical structure of PEEK’s mer unit.

In order to conduct a thorough chemical analysis of XPS spectra obtained for modified PEEK surfaces, first the reference material, i.e., non-modified PEEK, was examined. For the non-modified PEEK’s C1s and O1s bands registered, the model of peaks corresponding to its chemical structure (Figure 5) was developed. 

**Figure 6:**
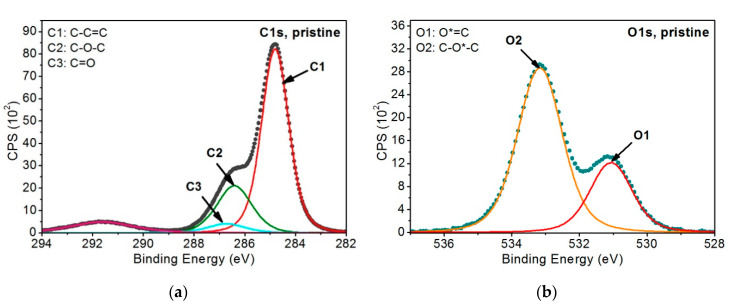
XPS spectra of non-modified PEEK: (**a**) C1s band, (**b**) O1s band.

Three peaks were modeled for the C1s band—Figure 6a. The first peak at 284.8 eV (FWHM 1.3 eV) represents the C–C=C structure in carbon rings (carbon atoms marked as 1 on Figure 5), the second peak at 286.4 eV (FWHM 1.6–1.7 eV) represents the C–O–C group (carbon atoms marked with 2 on Figure 5) and the third one at 286.7 eV (FWHM 1.7–1.8 eV) represents C=O (carbon atoms marked with 3 on Figure 5). Two peaks were modeled for the O1s band: the one at 531.1 eV (FWHM 1.5 eV) that represents the O*=C group and another one at 533.2 eV (FWHM 1.6 eV) that represents the C–O*–C group—Figure 6b. 

Carbon and oxygen bands in the model were related to each other so that the amount of oxygen single bonded with carbon was twice as little as the amount of carbon single-bonded with oxygen, which is in accordance with the chemical structure of the PEEK mer unit. A similar relation was set up for the peaks coming from carbon atoms double-bonded with oxygen, and the content of the peaks is equal, according to the material’s chemical structure. Moreover, *π* − π^*^ peak was introduced to the model, corresponding to the presence of carbon rings in the polymer’s structure. The model developed this way appropriately describes the unmodified PEEK.

The model developed was then used for the analysis of the spectra of the samples treated with EUV radiation and EUV radiation in the presence of nitrogen. Three variants of PEEK surface modification were used: (1) treatment of the PEEK surface with 40 and 150 EUV pulses, (2) treatment of the PEEK surface with 40 and 150 EUV pulses in the presence of nitrogen with density determined by the valve opening time of 300 µs, (3) treatment of the PEEK surface with 40 and 150 EUV pulses in the presence of nitrogen with density determined by the valve opening time of 350 µs. 

XPS examination of the first variant showed the presence of oxygen and carbon atoms as well as deep changes of the C1s and O1s peaks’ envelopes. For the second and third variants, apart from the presence of oxygen and carbon, the high content of nitrogen in the modified samples’ surface was observed, as a result of the interaction of ionized nitrogen on the PEEK surface.

**Figure 7:**
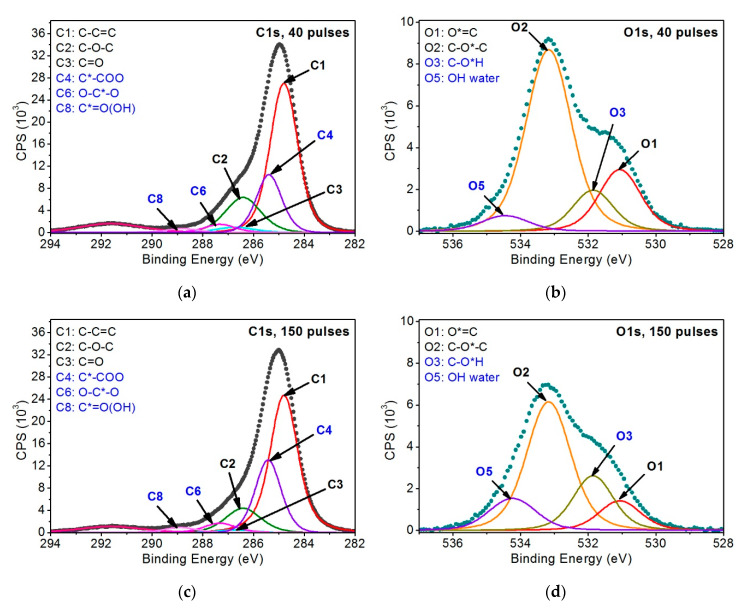
XPS spectra of modified PEEK: (**a**) with 40 EUV pulses—C1s band, (**b**) with 40 EUV pulses—O1s band, (**c**) with 150 EUV pulses—C1s band, (**d**) with 150 EUV pulses—O1s band.

Figure 7 shows the results of the XPS examination analysis for the first variant of the PEEK sample irradiated with 40 and 150 EUV pulses. 

Due to the dramatic change in the shape of the C1s and O1s peaks’ envelopes of the samples modified, not only peaks from the pristine PEEK model, but also other peaks describing changes of the polymer resulting from EUV irradiation, were added. Therefore, three additional peaks corresponding to functional groups were introduced to the C1s band, which probably formed during the breaking of bonds between atoms coming from polyetheretherketone mer units:

- C*–COO (C4)—at ~285.4 eV (FWHM 1.3–1.4 eV)—corresponds to secondary chemical shifts. This is most likely a result of breaking the bonds between atoms marked as 3 and 2 (Figure 5), linking vicinal carbon rings.
- O–C*–O (C6)—at ~287.3 eV (FWHM 1.4–1.5 eV)—is most likely the effect of breaking the bond between the carbon marked as 3 (Figure 5) and the carbon ring. The empty bond is then filled with an oxygen atom.
- C*=O(OH) (C8)—at ~288.8–288.9 eV (FWHM ~1.5 eV)—is most likely formed as a result of breaking the bond between the atom marked as 3 (Figure 5) and the carbon ring and then attaching the OH group to the carbon atom marked as 3.

As far as the O1s band is concerned, it was filled with the following peaks:

- C–O*H (O3)—at ~531.9 eV (FWHM 1.4–1.5 eV)—is most probably associated with the presence of OH groups within the chemical structure of the modified PEEK, as this peak corresponds to the peak C8 and fills the envelopes of the O1s band between O1 and O2 peaks modeled for the unmodified PEEK material.
- OH (water) (O5)—534.2–534.5 eV (FWHM 1.6–1.7 eV)—this peak most likely comes from OH groups of the water adsorbed on the sample surface after taking it out from the vacuum.

**Figure 8:**
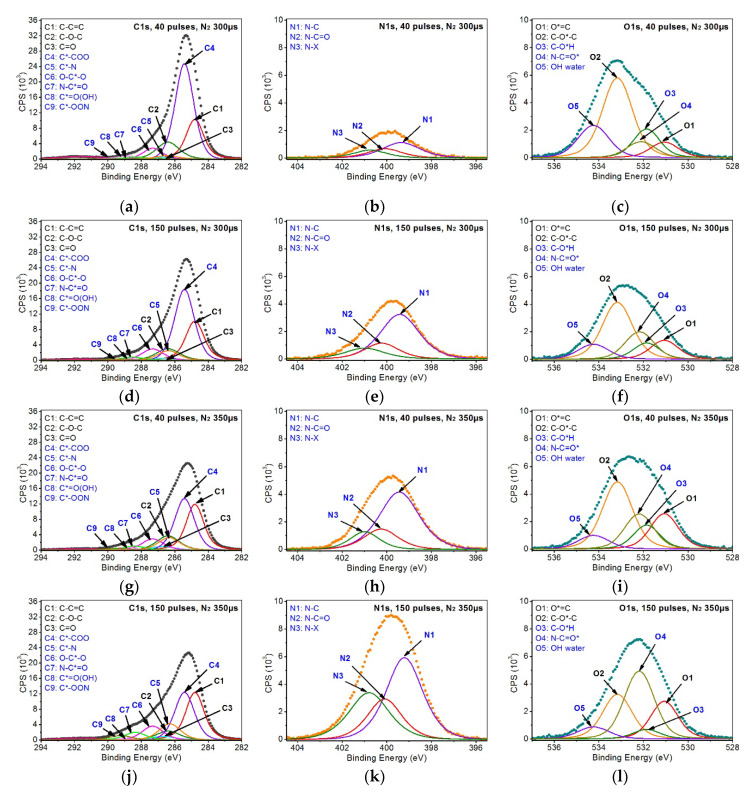
XPS spectra of modified PEEK: (**a**) C1s band for 40 pulses (N_2_ 300 µs), (**b**) N1s band for 40 pulses (N_2_ 300 µs), (**c**) O1s band for 40 pulses (N_2_ 300 µs), (**d**) C1s band for 150 pulses (N_2_ 300 µs), (**e**) N1s band for 150 pulses (N_2_ 300 µs), (**f**) O1s band for 150 pulses (N_2_ 300 µs), (**g**) C1s band for 40 pulses (N_2_ 350 µs), (**h**) N1s band for 40 pulses (N_2_ 350 µs), (**i**) O1s band for 40 pulses (N_2_ 350 µs), (**j**) C1s band for 150 pulses (N_2_ 350 µs), (**k**) N1s band for 150 pulses (N_2_ 350 µs), (**l**) O1s band for 150 pulses (N_2_ 350 µs).

The introduction of the peaks given above allowed for an appropriate filling of C1s and O1s bands’ envelopes of the samples modified. The analysis also assumed that the introduced peaks, which were taken from the pristine PEEK, would keep their parameters (i.e., peak position, FWHM). Additional peaks added were associated with the most probable changes in the chemical structures of the material exposed to EUV radiation. Regarding variants 2 and 3 of the sample modifications carried out using EUV radiation in nitrogen presence, it was ascertained that it is necessary to introduce another seven peaks to the C1s, O1s, N1s bands, related to the incorporation of nitrogen atoms to the PEEK surface (Figure 8):

- C*–N (C5)—at 286.3–286.4 eV (FWHM ~1.5 eV)—peak related to the incorporation of nitrogen atoms into the PEEK structure as a result of breaking bonds of the carbon marked as 2 or 3 (Figure 5) and introducing nitrogen atoms in that place.
- N–C*=O (C7)—at 288.4–288.5 eV (FWHM 1.5 eV)—is formed most likely as a result of carbon bond breaking (carbon marked as 3—Figure 5) and filling it with a nitrogen atom.
- C*–OON (C9)—at 289.3–289.5 eV (FWHM 1.5 eV)—due to a huge chemical shift of this peak relative to the C1 peak, it was assumed that this structure most probably contains two oxygen atoms and a single nitrogen atom. The proof for the presence of this peak is a huge change in the shape of the C1s’ envelope around the binding energy values of 289.3–289.5 eV. Such change was not observed for the samples treated with EUV radiation only. Taking into account the location of this peak on the energy axis and the fact that the oxygen content in the samples modified with EUV irradiation in the presence of nitrogen does not exceed the content in the samples modified with EUV irradiation alone, there is a low probability that this peak represents structures containing oxygen only.

- N*–C (N1)—at 399.2–399.4 eV (FWHM 1.9–2.1 eV)—peak corresponds to C5
- N*–C=O (N2)—at 400.0–400.2 eV (FWHM 1.8–1.9 eV)—peak corresponds to C7
- N*–x (N3)—at 400.8–400.1 eV (FWHM 1.8–2.1 eV)—it is difficult to interpret this peak unambiguously. However, taking into account the measurements of the reference polymers containing nitrogen atoms, such as PU, Kapton, nylon, confronted with the data taken from literature, it can be assumed that these are nitrogen atoms bonded with carbon atoms which form chemical bonds with at least two oxygen atoms.
- N–C=O* (O4)—at 532.1–532.2 eV (FWHM ~1.5–1.6 eV)—peak corresponds to C7.

The analysis of the samples modified with EUV radiation in the presence of nitrogen additionally assumed that the peaks in C1s and O1s bands would be tightly related to each other in terms of their percentage content. As a result, the content of the C5 peak is the same as the content of the N1 peak and the content of the C7 peak is equal to the content of both the N2 and O4 peaks as they are associated with the same chemical structures/functional groups. However, the N3 peak turned out to be much more difficult to interpret and, therefore, such an association could not be established. Nevertheless, the presence of this peak is certain, as indicated by the shape of the N1s band and the results of the analysis of the polymers containing nitrogen in their structure, which suggests that FWHM of the peaks in the N1s band should not exceed 2.1 eV. Taking the specificity of the modification process, it is highly probable that there can be even more peaks in the N1s band; however, the simplest model was developed for the analysis that properly reflects the chemical changes that took place.

Precise positions, FWHMs, and percentage contents of peaks from C1s, N1s, O1s bands for all the samples are listed in Table 1. 

The analysis assumed that the sum of all the peaks from all the bands is 100 at.% for a given sample.

As can be seen in Table 1, modification carried out leads to significant changes in the percentage content of functional groups coming from the pristine PEEK. The most explicit changes appear for the C1 peak, the percentage content of which lowers from 62.3 at.% for the pristine PEEK to 49.8 at.% and 50.0 at.% for the PEEK treated with 40 EUV pulses and 150 EUV pulses, respectively. Much lower percentage content of the C1 peak was observed for the samples modified with EUV radiation in the presence of nitrogen. This percentage content is from 19.6 at.% to 23.3 at.%. The percentage contents of C1 observed for this group of samples are close to one another but are not correlated with the number of pulses and the valve opening time. 

Similar changes in the percentage content were observed also for the chemical groups marked as C2 and C3 as well as for the peaks O1 and O2. In each of these cases modification leads to a decrease in the percentage content versus the pristine material. Their percentage content varies and depends on the parameters of a modification. However, similarly as in the case of C1, no explicit relation between the percentage content of the peaks, the number of pulses, and the valve opening time is observed.

Evident relation between the percentage contents and the parameters of modification is noticed for the samples irradiated with EUV in the presence of nitrogen. It is noticeable specifically for the C5, C7, and C9 peaks representing the chemical groups containing nitrogen atoms and correlated with the N1, N2, and N3 peaks in the N1s band as well as with the O4 peak in the O1s band in the model developed. Their percentage content increases with the increase in the number of pulses used and the valve opening time. In the N1s band, it is the N1 peak that has the highest percentage content in each sample modified. The maximum percentage content of this peak is noticed for the sample modified with 150 EUV pulses, for which the valve opening time is 350 µs. 

In order to correctly fill the envelope of the C1s and O1s band, the C4, C6, C8 and O3, O5 peaks were also introduced that represent the functional groups containing the carbon-oxygen bond. For the sake of the analysis, the minimum number of the peaks appropriately describing the C1s and O1s bands was assumed. However, due to the random nature of the formation of new functional groups on the PEEK surface, the model developed may not include all the chemical structures that may arise as a result of the modification applied. This is the reason for which certain randomness is observed in the percentage contents of the C1–C3 and O1–O2 peaks coming from the pristine material. 

Overall percentage contents of elements for the PEEK samples examined are listed in Table 2. 

As can be seen in Table 2, the pristine PEEK contains 86.1 at.% of carbon and 13.9 at.% of oxygen. Modification using only EUV radiation leads to a decrease in the percentage content of oxygen and therefore to an increase in the percentage content of carbon. An increase in the number of EUV pulses results in a decrease in the percentage content of oxygen to 11.7 at.% for 40 EUV pulses and 9.8 at.% for 150 EUV pulses. As described previously, this causes changes in C1s and O1s envelopes and the appearance of new functional groups.

As can be seen in Table 2, the method of modification using EUV radiation in the presence of nitrogen that was used enables incorporating a vast number of nitrogen atoms into the polymer surface. The percentage content of nitrogen atoms depends on both the number of pulses used (40, 150) and the opening time of the valve from which nitrogen was injected (300 µs, 350 µs). The percentage content of nitrogen obtained ranges from 3.5 at.% for EUV 40 (N_2_ 300 µs) to 17.2 at.% for EUV 150 (N_2_ 350 µs). As can be noticed, the increase in the percentage content of nitrogen is not directly proportional to the number of pulses. However, extending the time of nitrogen injection of 50 µs results in a more than doubled increase in the percentage content of nitrogen in the modified samples. A thorough analysis of the N1s band also shows that no matter what the modification parameters are (number of pulses, valve opening time), the same chemical structures/functional groups appear and only their percentage content varies. No explicit relation between modification parameters and the percentage content of peaks in the N1s band was observed, though. This can be the effect of the stochastic character of the chemical changes that take place on the surface of samples during the modification process.

As for the proportion of oxygen in these samples, it is lower than for the pristine PEEK and slightly lower than for the samples treated with the same number of EUV pulses in the absence of nitrogen. The percentage content of oxygen in these samples is very similar and shows no correlation with the change in nitrogen at.%. Theoretically, it would be expected that due to the modification the oxygen atoms would be replaced by nitrogen atoms. In fact, the percentage content of the nitrogen increases with the decrease in the percentage content of carbon. The final percentage contents of the oxygen for these samples, as well as for all the others, depends not only on the modification process itself, but also from the fact that just after the removal of the samples from the vacuum chamber, the empty bonds on the surface of the samples become saturated with oxygen atoms.

## 4. Conclusions

In this paper, the influence of EUV irradiation and the combination of EUV irradiation and low-temperature nitrogen plasma on the physico-chemical properties of PEEK polymer was presented. It was found that due to the modification, the topography of the PEEK surfaces significantly changed. The micro- and nanometer-scale features, including characteristic conical structures, appeared on the PEEK surface, and an increase in the average surface roughness was observed (from 16.2 nm for pristine PEEK up to 115 nm for modified PEEK). The modification with the use of EUV pulses alone, and the modification by the simultaneous interaction of the EUV pulses and the nitrogen photoionized plasma, also dramatically affected the chemical composition of PEEK surfaces. Modification using only EUV radiation led to a decrease in the percentage content of oxygen, which increased the percentage content of carbon, and the appearance of the new chemical groups. On the other hand, modification with the use of EUV radiation in the presence of nitrogen resulted not only in the appearance of new functional groups but in the incorporation of a vast amount of nitrogen up to 17.2 at.% as well. Therefore, the modification technique presented may be considered as a useful tool for surface activation of the PEEK polymer.

## Figures and Tables

**Table 1:** Binding energy range, FWHM range, and atomic concentration of different functional groups for pristine PEEK and modified PEEK.

Symbol of the Peak	Chemical Group	Position (eV)	FWHM (eV)	PEEK (at.%)	PEEK EUV40 (at.%)	PEEK EUV150 (at.%)	PEEK EUV40 N_2_ 300 µs (at.%)	PEEK EUV150 N_2_ 300 µs (at.%)	PEEK EUV40 N_2_ 350 µs (at.%)	PEEK EUV150 N_2_ 350 µs (at.%)
C1	C-C=C	284.8	1.3	62.3	49.8	50.0	19.6	21.3	23.2	22.5
C2	C*–O–C*	286.4	1.6–1.7	19.9	14.6	10.5	9.9	7.4	8.5	5.0
C3	C=O	286.7	1.7–1.8	4.0	2.3	1.1	0.9	1.2	2.1	2.0
C4	C*–COO	285.4	1.3–1.4	-	17.2	22.8	46.0	37.7	26.8	22.2
C5	C*–N	286.3–286.4	1.5	-	-	-	1.8	5.5	7.1	8.2
C6	O–C*–O	287.3	1.4–1.5	-	3.0	3.8	5.3	6.6	6.3	7.0
C7	N–C*=O	288.4–288.5	1.5	-	-	-	1.0	1.7	2.2	3.8
C8	C*=O(OH)	288.8–288.9	1.5	-	1.5	2.0	1.6	0.9	1.3	0.5
C9	C*–OON	289.3–289.5	1.5	-	-	-	0.0	0.6	0.9	2.2
N1	N*–C	399.2–399.4	1.9–2.1	-	-	-	1.8	5.5	7.1	8.2
N2	N*–C=O	400.0–400.2	1.8–1.9	-	-	-	1.0	1.7	2.2	3.8
N3	N*–x	400.8–401.0	1.8–2.1	-	-	-	0.8	1.4	1.6	5.2
O1	O*=C	531.1	1.5	4.0	2.3	1.1	0.9	1.2	2.1	2.0
O2	C–O*–C	533.2	1.6	9.9	7.3	5.3	5.0	3.7	4.3	2.5
O3	C–O*H	531.9	1.4–1.5	-	1.5	2.0	1.6	1.0	1.3	0.5
O4	N–C=O*	532.1–532.2	1.5–1.6	-	-	-	1.0	1.7	2.2	3.8
O5	OH(water)	534.2–534.5	1.6–1.7		0.6	1.4	2.0	1.0	0.9	0.7

**Table 2:** The atomic concentration of carbon, nitrogen, and oxygen for unmodified and modified PEEK.

Elements	PEEK (at.%)	PEEK EUV40 (at.%)	PEEK EUV150 (at.%)	PEEK EUV40 N_2_ 300 µs (at.%)	PEEK EUV150 N_2_ 300 µs (at.%)	PEEK EUV40 N_2_ 350 µs (at.%)	PEEK EUV150 N_2_ 350 µs (at.%)
C	86.1	88.3	90.2	86.0	82.9	78.4	73.3
N	-	-	-	3.5	8.6	10.9	17.2
O	13.9	11.7	9.8	10.5	8.5	10.8	9.5
